# Telemedicine and the environment: life cycle environmental emissions from in-person and virtual clinic visits

**DOI:** 10.1038/s41746-023-00818-7

**Published:** 2023-05-09

**Authors:** Cassandra L. Thiel, Natasha Mehta, Cory Sean Sejo, Lubna Qureshi, Meagan Moyer, Vincent Valentino, Jason Saleh

**Affiliations:** 1 NYU Langone Health, Departments of Population Health and Ophthalmology, New York, NY USA; 2 Stanford Department of Internal Medicine, Stanford, CA USA; 3 Stanford Health Care, Digital Health, Stanford, CA USA; 4 City of Seattle, Office of Economic Development, Seattle, WA USA; 5 Palo Alto Veterans Affairs & Stanford University, Stanford, CA USA

**Keywords:** Health services, Climate-change mitigation

## Abstract

Concern over climate change is growing in the healthcare space, and telemedicine has been rapidly expanding since the start of the COVID19 pandemic. Understanding the various sources of environmental emissions from clinic visits—both virtual and in-person—will help create a more sustainable healthcare system. This study uses a Life Cycle Assessment with retrospective clinical data from Stanford Health Care (SHC) in 2019–2021 to determine the environmental emissions associated with in-person and virtual clinic visits. SHC saw 13% increase in clinic visits, but due to the rise in telemedicine services, the Greenhouse Gas emissions (GHGs) from these visits decreased 36% between 2019 and 2021. Telemedicine (phone and video appointments) helped SHC avoid approximately 17,000 metric tons of GHGs in 2021. Some departments, such as psychiatry and cancer achieved greater GHG reductions, as they were able to perform more virtual visits. Telemedicine is an important component for the reduction of GHGs in healthcare systems; however, telemedicine cannot replace every clinic visit and proper triaging and tracking systems should be in place to avoid duplicative care.

## Introduction

Spending on healthcare typically accounts for a large proportion of a national budget, and this industry has also become one of the largest contributors to greenhouse gas (GHG) emissions^1,2^. As a result, there is now a substantial effort being made to understand and reduce GHGs within the industry^3–8^.

Telehealth-based care delivery has been identified as a possible tool in achieving these goals in carbon footprint reduction by removing the need for patients to burn fossil fuels through traveling to physical clinic locations^3,9–13^. These interventions also positively benefit chronic health outcomes that are affected by pollution, such as COPD and cardiovascular health, by reducing pollution^14^. In addition to reducing GHGs from patient travel, telehealth-based delivery has been shown to be cost effective, especially for patients who may have to request absences from work, replacements for care-giving activities, or arranging transportation^15–17^. Telehealth services can also increase access to care by reducing the barriers for patients, including total time, distance traveled, and effort required to seek medical care^18–20^. This novel style of practice has been widely adopted by clinicians, especially after the Centers for Medicaid and Medicare Services (CMS) established payment parity for telehealth services in the wake of the COVID-19 pandemic through the Consolidated Appropriations Act (2022)^21^. It also seems to be popular, demonstrating high patient satisfaction outcomes^22,23^.

Recent studies have shown a positive correlation between clinical care delivered via telehealth and a reduction in patient miles traveled to receive care^24^. However, these studies do not assess the environmental footprint of clinical care beyond travel or are narrowly focused on specialty practice or department. As CMS considers updates to their telehealth reimbursement policies, it is important to understand the wider implications of telehealth adoption. This study examines life cycle GHG emissions from telehealth services and in-person care delivery locations at a health system level, showing how the growth in telemedicine can both increase access to care and decrease a health systems’ carbon emissions.

## Results

### GHG Emissions from Clinical Visits

**Fig. 1:**
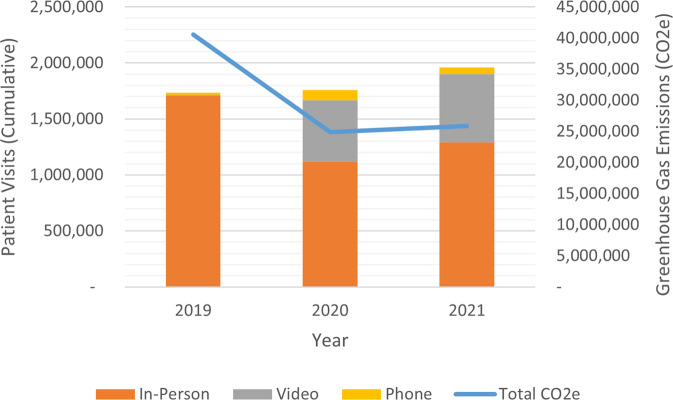
Number of Patient Visits and Cumulative Estimated GHG Emissions. This is modeled under baseline assumptions of minimal supplies, a 402 km cutoff for car travel, and WECC power grid in a 10′ × 10′ exam room consuming the SHC average energy per sf per minute.

Unsurprisingly, SHC saw a growth of virtual visits across the study period. Total visits increased 13% between 2019 and 2021, from 1,733,020 patient visits to 1,961,768 (Fig. 1). During this same time, SHC experienced an estimated 36% drop in their GHG emissions from clinic visits (assuming our baseline scenario with minimal supply utilization during each visit), from approximately 40,600 metric tons of CO2e to 25,900 tons. In 2021, SHC’s average in-person visit emitted an estimated 20 kg CO2e, while a phone-based virtual visit emitted only 0.02 kg CO2e and a video visit emitted 0.04 kg CO2e on average. In 2021, phone visits were conducted with 59,635 patients and video visits were conducted with 612,700 patients.

**Fig. 2:**
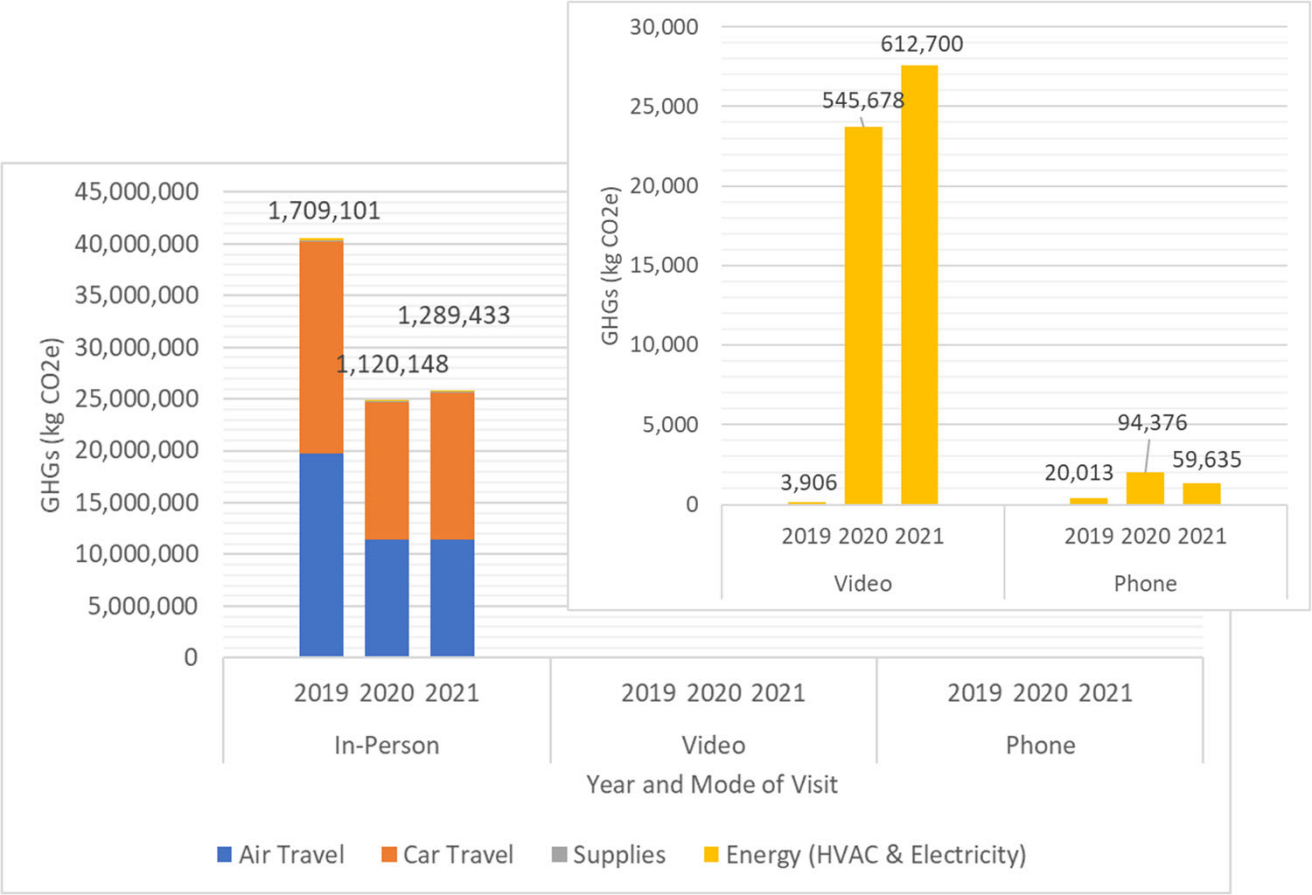
Total Greenhouse Gas (GHG) Emissions from SHC Clinical Visits by year and mode of visit. Colors signify sources of GHG emissions; Values above columns indicate total number of visits by this mode and year; Video and Phone telehealth visits are shown in pop-out box due to scale; HVAC heating, ventilation, and air conditioning.

Patient travel dominates GHG emissions sources for in-person visits (Fig. 2). In 2019, 49% of emissions were from assumed air travel and 51% from car travel. This shifted slightly by 2021, with 44% of emissions originating from an estimated 93,900,000 km of airline travel and 55% from 42,900,000 km of car travel.

Emissions from virtual visits were significantly less than in-person visits (Fig. 2). However, electricity used on computers and phones during virtual visits in 2021 still resulted in 29,000 kg CO2e, equivalent to burning 3252 gallons of gasoline or the energy usage of 3.6 homes for one year^25^.

The LCA results for other emissions impact categories (such as air pollution, toxicity, etc.) for SHC’s baseline case can be found in the Supplemental Information, Tables 5–7 and Fig. 6.

### Emissions avoided through Telehealth

**Fig. 3:**
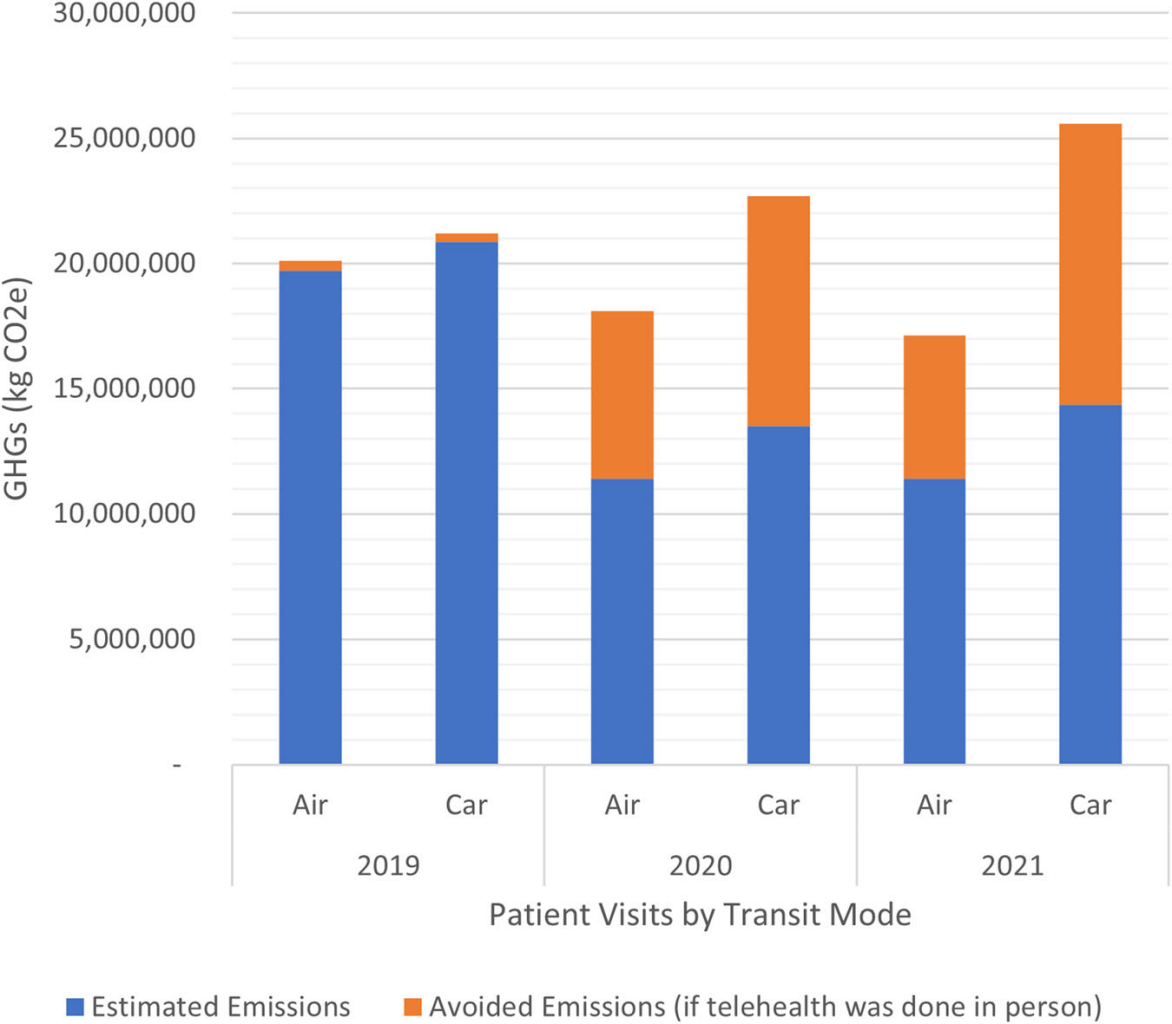
Avoided GHG emissions from televisits at SHC. GHG Greenhouse Gas, SHC Stanford Health Care.

With our assumptions around in-person visits, SHC’s virtual care system reduced their 2021 GHG emissions by nearly 17,000 metric tons (Fig. 3) as compared to treating those same patients in person. This is the equivalent of over 2100 homes energy use for a year or the CO_2_ sequestered by nearly 20,000 acres of US forest in one year^25^.

### Visits and emissions by department

**Fig. 4:**
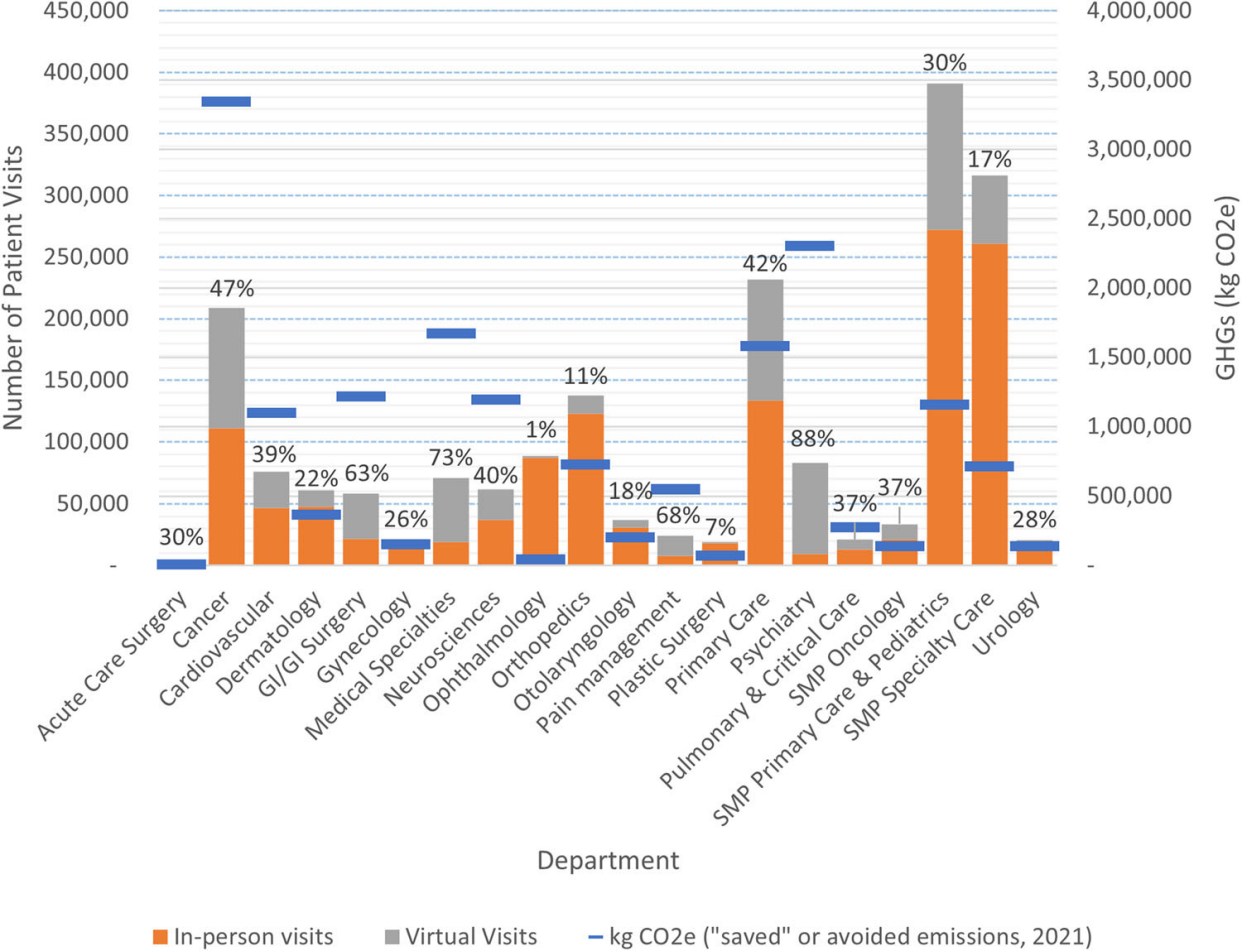
Avoided Greenhouse Gas (GHG) emissions and number of total visits by SHC Departmen in, 2021. The % values indicate % of total visits that were conducted virtually; SMP Stanford Medical Partners.

Some medical departments treat conditions that are more appropriate for telemedicine (Fig. 4). SHC saw the largest growth of telemedicine in departments such as psychiatry (where 88% of visits were virtual in 2021), medical specialties (73%), pain management (68%), GI surgery (63%), and cancer (47%). Unsurprisingly, certain specialties did not see large increases in telemedicine, including ophthalmology (1% of visits were virtual in 2021), plastic surgery (7%), orthopedics (11%), and otolaryngology (18%).

Emissions per patient ranged by department and by visit type. For in-person visits, primary care and pediatrics emitted the least per visit at 7.33 kg CO2e/visit, suggesting their patients travel least. Orthopedics had the largest per visit emissions at 63.8 kg CO2e/in-person visit, see SI Table 4. Per visit, virtual medicine emits less than 1% of the GHGs of an in-person visit, with a range of 0.02 to 0.08 kg CO2e per visit, depending on the department.

### Sensitivity analyses

We conducted sensitivity analyses using SHC’s 2021 data. The assumed mode of patient travel has the largest impact on our model. When we modeled all patient travel as occurring via passenger car, in-person visit emissions increased 77%, from 25,700 metric tons to 45,400 metric tons. For in-person visits, energy sources had little influence on emissions outcomes. Solar power reduced modeled emissions slightly and the US average grid mix increased emissions slightly. Changing the assumed energy intensity of the clinics for the in-person visits, or for the in-person visits avoided by virtual visits, did not change the outcomes. Transportation of patients dominates SHC’s per-visit emissions.

For virtual care specifically, changes to energy sources did impact modeled emissions, with solar reducing virtual visit emissions nearly 70% and the US grid mix leading to a 20% increase in estimated telehealth emissions (See Supplementary Information, Table 6). A maximum supply list, though unrealistic for most clinical visits, increased total GHG emissions from all in-person 2021 visits by about 1.1% or 277,000 kg CO2e.

Modes of transportation will change study results, with the large caveat that access to various modes of transit are limited. For example, an aircraft would be useless for short-distance travel, a bike useless for long-distance, an appropriate bus route may not be accessible, or a car may be unaffordable. Therefore, this analysis speaks only to theoretical changes to emissions rather than practical changes. See SI for more information.

## Discussion

Comparing 2019 to 2021, our institution conducted 13% more visits while reducing GHG by 36% (from 40,600 metric tons of CO2e to 25,900 metric tons of CO2e). For in-person visits, transportation is the major driver of GHG and accounts for the greatest savings when converted to virtual visits. Overall, virtual visits at SHC emit less than 1% of the GHG emitted by in-person visits (0.02 kg CO2e per virtual visit compared to 20 kg CO2e per in-person visit). Additionally, we were able to track specialty differences to show variable practicality of virtual visit adaptation. Our study expands on previous research by including a more comprehensive assessment of factors contributing to GHG emissions. Importantly, many studies fail to account entirely for transportation, contributions of PPE and equipment, electricity costs, and space necessary for patient care^24^.

There is a wide range in the purported reduction in GHG emissions from virtual (telephone visits and video visits) compared to in-person visits at 0.69–893 kg CO2e reduced per patient visit^16,26^. This reported variability is due to differences in research methods, study boundaries or the type of telehealth being analyzed, and a variety of other factors. Prior literature shows the leading culprit of GHG emissions from in-person visits is transportation^3,4,9,10,12,24,26^, which is consistent with this study. However, many other factors contribute to GHG emissions, including PPE, supplies, and electricity/energy cost^9,16,26^. In one large study of Greek miliary hospitals, electricity consumption was found to be the largest contributor of GHG emissions, and one of the proposed solutions was decarbonization of the energy sector^3^. Furthermore, there is considerable GHG emissions from the production and transportation of surgical equipment^4^.

Our study displays this heterogeneity within the literature by revealing the variability in factors that contribute to an excess of GHG emissions within different locations (rural versus urban) and different specialties (especially surgical versus nonsurgical)^12,16,24^. This example is highlighted in one study suggesting virtual visits may become the “greener” option within only a few kilometers distance between clinic and patient when compared to patient transport by car^9^. Similarly, our study found primary care and pediatric admissions account for the least CO2e per visit relative to other specialties (7.33 kg CO2e/visit), which is likely due to the relative proximity between patients and their primary care physician.

It is likely that the wide adoption of telemedicine will carry with it a significant reduction in overall GHG emissions associated with the delivery of care. As with many aspects in medicine, implementation of a new model for patient care will need to confer equivalent or added convenience, cost and clinical effectiveness when compared to the standard of care: in-person visits. These benefits should exist for both the patient and the provider.

Interestingly, health care utilization and disease burden increase with increasing distance between patients and their PCP, especially with patients going to academic centers^27^. Telemedicine appears to decrease cancellations and no-show appointments, leading to more patients completing appointments^28^.

Multiple studies have demonstrated a high level of patient satisfaction with telemedicine, especially since the start of the COVID-19 pandemic^29,30^. Though these visits are generally viewed positively by most patients, they still frequently show lower satisfaction rates than face-to-face encounters^31^. This may be due to a desire for a more detailed exam as well as difficulty relaying symptoms^30^. It is likely that as telemedicine encounters become a standard offering, patient satisfaction will improve to the point where it is on par with traditional visits.

Another barrier to telehealth implementation is cost. For some practice settings there will be an upfront cost to acquire equipment for telehealth as well as a period of lower efficiency while transitioning to a different model of patient visits, but there is likely to be an eventual net cost savings^32^. Traditional visits carry a large opportunity cost in terms of missed work, travel time and missed recreation for patients^33^. Based on the amount of time spent traveling and waiting to see a physician, telehealth has the potential to decrease these costs dramatically^34^.

A successful virtual visit is perhaps the greatest factor when optimizing cost, patient satisfaction, convenience, and reduction in GHG emissions. A successful virtual visit is a visit resulting in a diagnosis and plan of care without the need for an additional in-person visit with the same provider for the same reason. Though conversion rates are variable, nearly all medical settings for telehealth will have some rate of failure which then requires an office visit. This may be due to need for more detailed physical exam, lab work, radiographs or even patient preference. We can logically conclude that these failures of telemedicine result in greater overall cost, less convenience and greater GHG emissions since the patient will need a virtual and a face-to-face visit rather than one or the other. The ability to accurately predict which telehealth visits are likely to be successful will lead to greater rates of adoption and greater benefits. We did not attempt to estimate virtual care failure rates in this study, though it would be interesting for future work.

It is a reasonable assumption that most of the virtual visits in this study were successful visits despite there being few guidelines or guidance in this area. Given Palo Alto is largely a suburban area, there is likely little draw for leisure or shopping. Another example is that we may overestimate transportation from persons who, for example, live far away but work close to the clinic. In these scenarios, the small detour to clinic from their place of work may be negligible. However, these factors are variable from person-to-person and would be considerably difficult to account for.

Some specialties may lend themselves naturally to televisits while others may require a more traditional model of face-to-face visits for the physical exam. Different specialties may carry varying levels of reliance on the physical exam leading to higher telemedicine appointment failure rates. In a recent study of orthopedic patients during the pandemic, 46% of patients required conversion to in-person visits^30^. Another study demonstrated a high level of concern on the part of spine surgeons due to inability to perform an in-person exam^22^. Each provider will have their own level of comfort making a diagnosis in a virtual setting. The solution to the quandary is individualized studies by specialty and by diagnosis to determine which patients are likely to have a successful virtual visit and which are likely to need an additional in-person visit, potentially negating most or all of the benefits of telemedicine. Additionally, providers may choose to study telehealth in their own practice to determine how best to apply it to their specific patient population and practice style.

In addition, different patient populations will have different comfort levels with computer applications, potentially lowering success and patient satisfaction. Some populations with less access to technological resources or less comfort or ability to use those resources, may prefer a virtual visit by telephone only. However, CMS will no longer be reimbursing these visits at parity with in-person visits, disincentivizing providers from using this approach^20,35^. Alternatively, patient communities in more rural settings may be more enthusiastic about telehealth’s beneficial ability to drastically decrease travel time, travel distance, and associated GHG emissions. We should also take the patients’ wishes into account when deciding on a visit format. If a patient is firmly against a virtual visit, we may do them and ourselves a disservice by requiring it. This may result in lower patient satisfaction or a reduction in quality of care.

Policy changes seen with adapting to the COVID-19 Pandemic, including expanding eligibility for reimbursement of many telehealth visits^36^, provide a pathway and precedent for reform. This manuscript serves to further demonstrate the potential environmental benefits of virtual visits, and the need to re-evaluate and expand policies for reimbursement of these services. The potential future policy implications of this paper expand beyond the United States. These findings will likely be replicated in other nations with similar access to car and airplane travel, and similar access to primary care and subspecialty physicians. However, there will likely be differences in GHG savings in areas with well-established and robust public transport systems.

Finally, while adoption of telehealth services drastically reduces the emissions associated with an individual visit, telehealth does not achieve the zero-emissions status required to ameliorate the worst of climate change. Without decarbonization of electric grids, telehealth visits are likely to increase in energy intensity as broadband and high-speed internet infrastructure expands to more regions in the US^37,38^. Video appointments will be higher quality, but technically less climate friendly. This is to say, the use of telehealth or telemedicine and the expansion of broadband infrastructure should continue to be supported and encouraged. Simultaneously, policies need to be enacted to further decarbonize their use.

Excluding virtual visits’ HVAC emissions is a major but necessary omission. A provider joining a virtual call is likely to be in a conditioned space, either in the clinic or in their home. This space may be shared with other individuals or used for multiple activities, making the allocation of HVAC emissions challenging. A patient could join a virtual call from anywhere, including unconditioned spaces such as the outdoors, a vehicle, or conditioned spaces such as a workplace, home, or any other building. The room size, HVAC settings, occupancy levels, and alternate uses are all factors in the allocation of a patient’s HVAC to their virtual visit emissions. Theoretically, everyone should be joining the virtual visit from within the state of California, as that is where medical licensing is most likely held; however, even within California, varying energy sources exist with different emissions profiles. There is another question of whether the space used by the provider and patient in a virtual visit would have been conditioned regardless of their occupying the space. For example, a patient joining from home would likely produce the same emissions from their home HVAC whether they attended the visit in person or virtually. All of these factors will change the emissions profiles and potential savings from clinical visits but are difficult to model with any accuracy given the available datasets.

The healthcare industry has a significant environmental impact on our planet due to the requirement of supplies, consumption of energy, and production of large-scale waste. As more focus and efforts are put on industry-wide reductions in environmental impact, it is important for the healthcare sector to recognize its responsibility to take appropriate action. The delivery of telemedicine services when appropriate, can help healthcare organizations deliver care that is also healthier for the planet.

## Methods

### Case location

Stanford Health Care (SHC) is the Stanford University affiliated academic medical center and health system headquartered in Palo Alto, California and is the only level one trauma center between the cities of San Francisco and San Jose. SHC serves patients in the Bay Area and beyond by offering inpatient hospital services, outpatient primary care and specialty health centers, physicians’ offices, virtual care offerings and health plan programs. Stanford Health Care operates one hospital, with 605 licensed beds, including 101 licensed intensive care unit beds. SHC and its affiliated outpatient services organization, Stanford Medical Partners (SMP) operates a total of 65 clinics across the Bay Area. During the COVID-19 pandemic, many clinics were closed to prevent the spread of infection, as a result, virtual visits became popular across many specialty areas. Stanford Health Care’s Digital Health Care Integration team supports clinical care teams who use telehealth and other digitally based care delivery modalities to enhance care access and health outcomes for our patients. Stanford Health Care’s Sustainability Project Office advocates and supports activities that reduce the environmental footprint of our organization’s operations and costs while improving the well-being of our staff, visitors, patients, and community. In June 2022, Stanford Medicine signed the Biden administration’s health care sector pledge to address climate change, promising to reduce climate-warming emissions by 50% by 2030 and achieve net-zero emissions by 2050.

This study uses environmental Life Cycle Assessment (LCA) to quantify the environmental emissions associated with a virtual and an in-person clinical visit across specialty areas offered at Stanford Health Care. LCA is conducted in 4 steps, according to ISO 14040 standards: (1) goal and scope definition, (2) life cycle inventory (LCI), (3) impact assessment, and (4) interpretation and analyses^39^. As this study was initially for quality improvement and resulting data was de-identified for analysis, this study did not require ethical approval. Participant informed consent was not sought, as data were retrospective, deidentified, and aggregated, making identification of individual participants impossible.

### Goal and scope definition

The functional unit for this study is one clinic visit, conducted either in-person or virtually. This study also estimates emissions from the total number of clinic visits occurring at SHC annually, from 2019 to 2021. Here, we will report GHG emissions in units of kg CO_2_-equivalents and metric tons of CO2-e. Results from other emissions categories are reported in Supplementary Information Table 1.

**Fig. 5:**
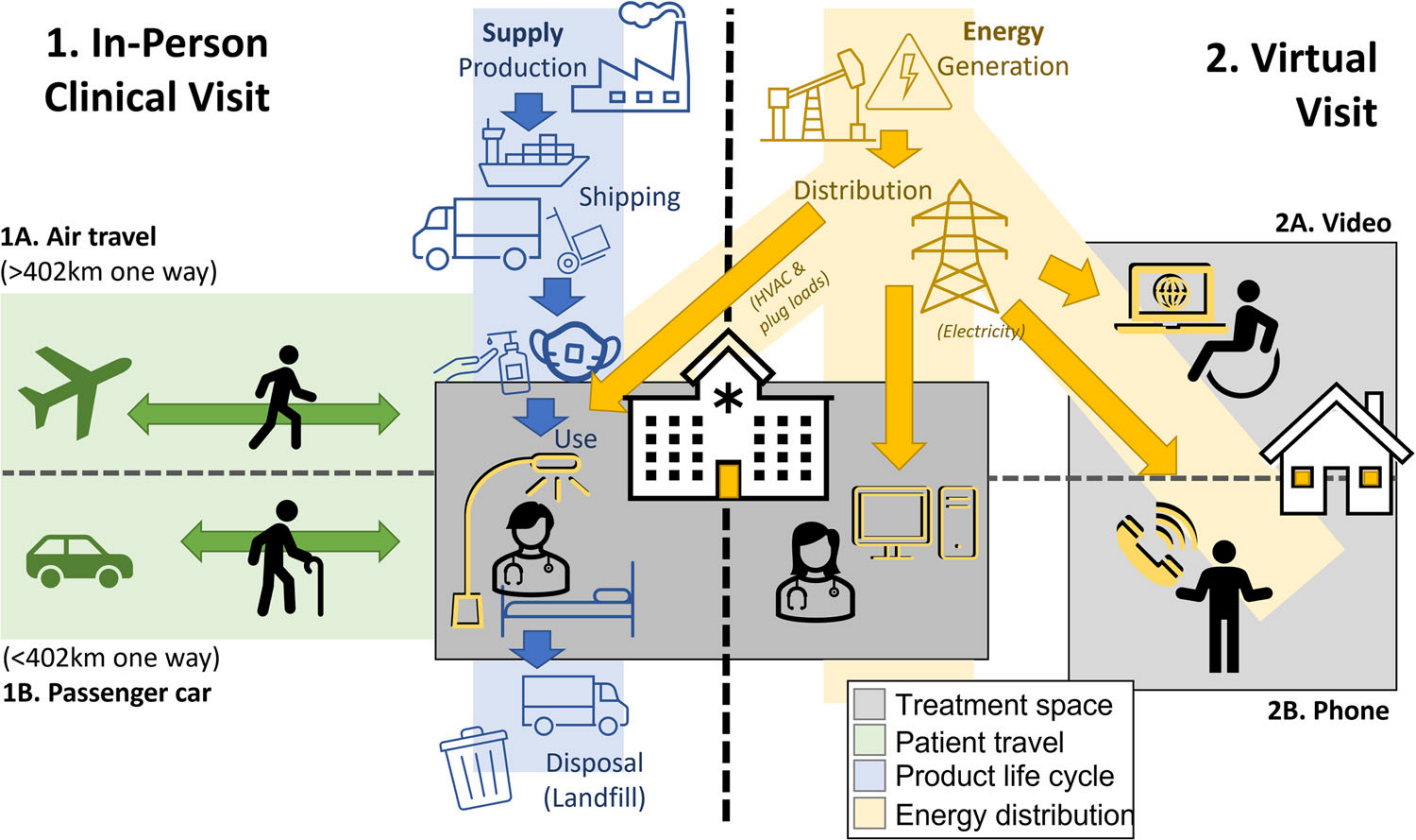
Flow diagram of the inputs for modeling the environmental footprint of in-person and virtual clinic visits at the study location. HVAC Heating, Ventilation, and Air Conditioning.

For in-person visits, patients must travel to the clinic, where they wait in a waiting area and are then escorted to a private exam room. Prior to their doctor’s visit, a nurse will often have the patient complete a digital questionnaire and (additionally, in office), will collect some data on the patient’s health, including blood pressure readings, height, and weight, depending on the specialty. For the virtual visit, the patient connects with a single clinician either by video conferencing or by telephone without video. For in-person visits, we include patient transportation to and from the clinic; the energy used in heating, ventilation, and air conditioning (HVAC) and lighting the exam room; and the supplies used, and waste generated, including PPE worn by clinicians (assumed to be one surgical mask per 10 patient visits), one paper-based exam table cover, one pump of hand sanitizer, and a sanitizing wipe (Fig. 5). For the virtual visit, we include the electricity needed to power a cellular phone for a call or the power needed for running video conference software. We also include the electricity use of the clinician, who we assume joins the virtual visit through a desktop computer in the clinic.

The clinician is highly likely to be in an indoor space with HVAC and lighting; however, the size of that space and the resulting energy usage is difficult to determine; therefore, we excluded it from our base assessment (see sensitivity section below for more information). We have excluded the energy needs for in the patient’s space, as the patient may be conducting their virtual visit from any location, including spaces without HVAC or lighting (such as outdoors) or a shared space (where energy usage could be allocated to other individuals or activities). Also excluded from this study is the commuting of staff for either visit, given the difficulties of allocating a staff member’s entire commute to a single visit. This is especially challenging for the virtual visit, where a clinician may not be commuting at all.

We assume that all virtual visits were appropriate, meaning that they did not convert to an in-person visit. This may not be accurate. We did not attempt to estimate how many of the in-person visits were eligible to be conducted virtually.

### Data sources and assumptions

Stanford Health Care’s Digital Health Care Integration team collected data on the number of in-person, phone, and video visits across SHC, by department, from 2019 through 2021. These data included the number of visits to each department and the duration of each visit in minutes, shown in SI Table 2. Though we did not collect exact data on the mode or distance traveled by patients, we calculated the average distance between the patient’s ZIP code and the ZIP code of the clinic visited. Any one-way distance greater than 250 miles (402 km) was assumed to be taken via airplane, while anything less than this value was assumed to be traveled by passenger car. The round-trip distance per patient was used to estimate emissions.

A list of basic supplies typically found in more patient visits was generated by the study team. These include one surgical mask, worn by the care provider for an estimated 10 cases; a serving of hand sanitizer; tissue to cover the exam table; and a sanitizing wipe to clean the exam room after the patient’s visit. The material components of each product were directly measured or estimated from literature. We assumed all products were manufactured in China and shipped approximately 3000 km by boat. We estimated a 40 km distance for distribution by freight truck before arrival at the clinic. All products were disposed in a sanitary landfill approximately 40 km from the clinic by truck. Of note, distances may vary by the exact clinic location, thus an average was assumed.

SHC’s engineering team provided record of electricity, gas, steam, and chilled water consumption across SHC’s clinics in 2020. These values were divided by the total surface area of the clinical space represented in the data and the number of minutes in a year (assumed to be 525,600 though this may artificially reduce the actual energy intensity of a clinical visit, as it assumes lights and equipment are drawing electrical power after hours). We then multiplied this ‘energy intensity’ by the floor area of an exam room, assumed to be a 10′ × 10′ space (or 9.3 m^2^), and the duration of the clinic visit, as captured in the medical records. As some buildings consumed varying quantities of energy, we included these ranges in one of our sensitivity analyses, described below.

### Life cycle inventory and impact assessment

To estimate the life cycle emissions of clinic visits, we use LCA software SimaPro 9.3.0.2^40^ and LCI database Ecoinvent v3.8^2^ with the allocation, cut off by classification approach. Ecoinvent is one of the most comprehensive LCI databases and is commonly used for healthcare LCAs. A list of model inputs and their assigned unit processes can be found in the Supplemental Information, Table 1. The impact assessment was conducted using the US Environmental Protection Agency’s TRACI 2.1 v1.06/ US 2008 (Tool for Reduction and Assessment of Chemicals and Other Environmental Impacts)^41^.

### Calculating reduction in GHGs from virtual visits

To estimate the amount of GHGs NOT emitted as a result of virtual visits, we estimated the GHGs of these visits, had they been in person. This would therefore add transportation of the patient, following the above assumptions for distance and mode, supplies production and disposal, and a shift in the amount of electricity used during the visit, which was done by assuming the number of minutes on the phone or video call would instead be the number of minutes spent consuming the average energy in a 10′ × 10′ exam room.

### Sensitivity analyses

Like most LCAs, our study makes many assumptions. To better understand the impact of our assumptions and to account for variability in telemedicine approaches at different institutions, we conducted multiple sensitivity analyses of our model inputs during the year 2021, shown in SI Table 2. To better understand the estimated 402 km cutoff for airplane travel, we ran an assessment where all patients were assumed to travel by car (Scenario “car”). Energy consumption of in-person visits (HVAC, etc.) is also variable, through differences in exam room size, building energy intensity, and energy sources. We utilized a max/min sensitivity approach to model this potential variability, using the minimum and maximum energy intensity by energy source from existing SHC clinics and assumptions on the minimum and maximum exam room size, shown in SI Table 3. Finally, we analyze the effect of different electric grid mixes on the outcomes of both in-person and virtual visits. Our baseline model uses the Western Electricity Coordinating Council (WECC) grid mix, and out sensitivity analysis assesses sourcing electricity from solar panels in the WECC region (which is most similar to our case study location) and from the average US grid mix, which is likely to have greater GHG emissions due to more carbon-intensive electric generation but may be more relevant to other healthcare sites. Though potentially less useful, we also modeled the impacts of a heftier supply list for in-person visits, assuming the use of one pair of exam gloves, two disposable gowns (one each for the provider and patient), hand sanitizer, a sanitary wipe, a surgical mask, and a table cover. It is unlikely that this is a normal practice in most exam spaces, especially for visits that could be conducted virtually.

Given initial results and the importance of patient’s transportation mode, we also conducted a sensitivity analysis exploring the impact of transportation mode on in-person visit emissions for 2021. The estimated total distance traveled by all in-person visits is 136,789,932 km. We use this as the input for a variety of transit modes, including bicycles, bus, and passenger cars with different fuel sources, as available in Ecoinvent v3.8.

### Reporting summary

Further information on research design is available in the Nature Research Reporting Summary linked to this article.

## Supplementary information

Supplemental Information

Reporting Summary

## Data Availability

Data in de-identified form may be made available from the corresponding author upon reasonable request.
