# Supplementary material for: Telemedicine and the environment: life cycle environmental emissions from in-person and virtual clinic visits
Source: NPJ Digit Med. 2023 May 9;6:87. doi: 10.1038/s41746-023-00818-7 (PMC10169113; doi:10.1038/s41746-023-00818-7)
Supplement: Supplementary file 1 — Supplemental Information [file 41746_2023_818_MOESM1_ESM.pdf]

Supplemental Information

Title: Telemedicine and the Environment: Life Cycle Environmental Emissions from In-Person and Virtual Clinic Visits

Supplemental Table 1: System inputs and unit processes used; including alternate scenarios for supplies. GLO=global, UP=unit process, U=unit, RoW=Rest of World, RER=Europe, CN=China, US=United States, WECC= Western Electricity Coordinating Council

**Transit Mode**	**amount (2021 total)**	**unit**	**Ecoinvent v3 UP**	**Source**		
Air travel	140921810.7	people-km	Transport, passenger aircraft, short haul {GLO}| transport, passenger aircraft, short haul | Cut-off, U	Patient travel estimated from ZIP code of address vs. clinic; distances over 400km assumed as flight mode		
Car travel	76428101.73	km	Transport, passenger car {RoW}| market for | Cut-off, U	Patient travel estimated from ZIP code of address vs. clinic; distances over 400km assumed as flight mode		
**Minimal Supply List**	**amount (per visit)**	**unit**	**Ecoinvent v3 UP**	**Source**		
Surgical Mask	11.7	g	Polypropylene, granulate {GLO}| market for | Cut-off, U	Direct measurement; allocated over total use (1/10th the production and disposal)		
	0.24	g	Cellulose fibre {RoW}| market for cellulose fibre | Cut-off, U	Direct measurement; allocated over total use (1/10th the production and disposal)		
	0.02925	g	Aluminium alloy, metal matrix composite {GLO}| market for | Cut-off, U	Direct measurement; allocated over total use (1/10th the production and disposal)		
	0.02925	g	Polyurethane, flexible foam {RoW}| market for polyurethane, flexible foam | Cut-off, U	Direct measurement; allocated over total use (1/10th the production and disposal)		
	0.01	kWh	Electricity, high voltage {CN}| market group for | Cut-off, U	Estimate; allocated over total use (1/10th the production and disposal)		
Hand Sanitizer	2.55	g	Benzyl alcohol {RER}| production | Cut-off, U	Estimate		
Table Cover	31.99347	g	Tissue paper {RER}| production | Cut-off, U	Estimate		
Sanitary Wipe	0.0212	g	Ammonium chloride (GLO) market for/cut-off, U	Duffy, et al		

	2.3	g	Isopropanol {RoW}| market for isopropanol | Cut-off, U	Duffy, et al		
	1	g	Fibre, cotton (GLO) market for/Cut-off U	Duffy, et al		
**Minimal Supply List - Transportation**	**amount (per visit)**	**unit**	**Ecoinvent v3 UP**	**Source**	**Assumed Distance (km)**	**Data Sources**
Shipping (from China)	0.117213	tkm	Transport, freight, sea, container ship {GLO}| market for transport, freight, sea, container ship | Cut-off, U	Estimate	3000	Estimates/assumptions - Google maps
	0.00156284	tkm	Transport, freight, lorry >32 metric ton, euro4 {RoW}| market for transport, freight, lorry >32 metric ton, EURO4 | Cut-off, U	Estimate	40	Estimates/assumptions - Google maps
Waste transit	0.00156284	tkm	Municipal waste collection service by 21 metric ton lorry {GLO}| market for | Cut-off, U	Estimate	40	Estimates/assumptions - Google maps
Waste scenario	39.071	g	Municipal solid waste (waste scenario) {US}| Treatment of waste | Cut-off, U	Calculated from measured data		
**Intensive Supply List (above, 1 mask per visit, PLUS:)**	**amount (per visit)**	**unit**	**Ecoinvent v3 UP**	**Source**		
Exam gloves (2)	9.6	g	Polybutadiene {RER}| production | Cut-off, U	Primary - weighed item; assumed material type		
Gown	110	g	Polypropylene, granulate {RER}| production | Cut-off, U + Electricity, high voltage {CN}| market group for | Cut-off, U	Primary - weighed item; assumed material type; energy from literature (secondary source)		
	0.5	kWh	Electricity, high voltage {CN}| market group for | Cut-off, U	Estimate		

Patient Gown	110	g	Polypropylene, granulate {RER}| production | Cut-off, U + Cellulose fibre {RoW}| market for cellulose fibre | Cut-off, U + Aluminium alloy, metal matrix composite {GLO}| market for | Cut-off, U + Polyurethane, flexible foam {RoW}| market for polyurethane, flexible foam | Cut-off, U	Primary - weighed item; assumed material type; energy from literature (secondary source)		
	0.5	kWh	Electricity, high voltage {CN}| market group for | Cut-off, U	Estimate		
**Intensive Supply List - Transportation**	**amount (per visit)**	**unit**	**Ecoinvent v3 UP**	**Source**	**Assumed Distance (km)**	**Data Sources**
Shipping (from China)	0.838413	tkm	Transport, freight, sea, container ship {GLO}| market for transport, freight, sea, container ship | Cut-off, U	Estimate	3000	Estimates/assumptions - google maps
	0.01117884	tkm	Transport, freight, lorry >32 metric ton, euro4 {RoW}| market for transport, freight, lorry >32 metric ton, EURO4 | Cut-off, U	Estimate	40	Estimates/assumptions - google maps
Waste transit	0.01117884	tkm	Municipal waste collection service by 21 metric ton lorry {GLO}| market for | Cut-off, U	Estimate	40	Estimates/assumptions - google maps
Waste scenario	279.471	g	Municipal solid waste (waste scenario) {US}| Treatment of waste | Cut-off, U	Calculated from measured data		
**HVAC Energy Use**	**amount (per min)**	**unit**	**Ecoinvent v3 UP**	**Source**		
Natural gas, heating	0.010331205	MJ/min	Heat, central or small-scale, natural gas {RoW}| market for heat, central or small-scale, natural gas | Cut-off, U	Direct measurement; averaged across all measured facilities		
Electricity, all uses plus chilled water	0.00625289	kWh/min	Transport, passenger car {RoW}| market for | Cut-off, U	Direct measurement; averaged across all measured facilities		
Steam from district heating	0.011718687	MJ/min	Video, one minute, WECC grid	Direct measurement; averaged across all measured facilities		

**Other Energy Use**	**amount (per min)**	**unit**	**Ecoinvent v3 UP (modified to use the WECC grid mix)**	**Source**		
Phone, electricity (patient)	2.17E-05	kWh/min	Electricity, low voltage {WECC, US only}| market for | Cut-off, U	Nurminen, 2010; assumed to be a cell phone		
Phone, desktop (clinician)	1	min	Operation, computer, desktop, office use {WECC}| market for | Cut-off, U			
Video, internet (patient)	1	min	Internet access, videoconference WECC, 0.7 Mbit/s {GLO}| market for | Cut-off, U			
Video, desktop (clinician)	1	min	Operation, computer, desktop, office use {WECC}| market for | Cut-off, U			

Supplemental Table 2: Model inputs for baseline scenario and sensitivity analyses, shown for 2021 only

Inputs for Sensitivity Analyses		*In-person visits (2021 cumulative)*					*Virtual visits (2021 cumulative)*	
**Scenario**	**Setup**	**Supplies(# visits)**	**Trash (# visits)**	**Car (km)**	**Aircraft (km)**	**HVAC (min)**	**phone min**	**video min**
Baseline (min supplies, WECC)	2021 In-person, air	39,213	39,213	0	93,908,607	882,760	0	0
	2021 phone, air (IF in person)	1,396	1,396	0	3,070,530	43,025	43,025	0
	2021 video, air (IF in person)	21,895	21,895	0	43,942,674	764,470	0	764,470
	2021 In-person, car	1,250,220	1,250,220	42,881,325	0	32,426,310	0	0
	2021 phone, car (IF in person)	58,239	58,239	2,560,264	0	1,595,630	1,595,630	0
	2021 video, car (IF in person)	590,805	590,805	30,986,513	0	18,729,540	0	18,729,540
	2021 - phone						1638655	
	2021 - video							19494010

Transportation: All car	2021 In-person, all car	1,289,433	1,289,433	136,789,932	-	33,309,070		
	2021 Phone (IF all car)	59,635	59,635	5,630,794	-	1,638,655		
	2021 Video (IF all car)	612,700	612,700	74,929,186	-	19,494,010		
Energy: WECC + solar	2021 In-person, air	39,213	39,213	0	93,908,607	882,760		
	2021 phone, air (IF in person)	1,396	1,396	0	3,070,530	43,025		
	2021 video, air (IF in person)	21,895	21,895	0	43,942,674	764,470		
	2021 In-person, car	1,250,220	1,250,220	42,881,325	0	32,426,310		
	2021 phone, car (IF in person)	58,239	58,239	2,560,264	0	1,595,630		
	2021 video, car (IF in person)	590,805	590,805	30,986,513	-	18,729,540	**phone min**	**video min**
	2021 - phone	-	-	-	-	-	1,638,655	-
	2021 - video	-	-	-	-	-	-	19,494,010
Energy: US Average	2021 In-person, air	39,213	39,213	0	93,908,607	882,760		
	2021 phone, air (IF in person)	1,396	1,396	0	3,070,530	43,025		
	2021 video, air (IF in person)	21,895	21,895	0	43,942,674	764,470		
	2021 In-person, car	1,250,220	1,250,220	42,881,325	0	32,426,310		
	2021 phone, car (IF in person)	58,239	58,239	2,560,264	0	1,595,630		

	2021 video, car (IF in person)	590,805	590,805	30,986,513	-	18,729,540	**phone min**	**video min**
	2021 - phone	-	-	-	-	-	1,638,655	-
	2021 - video	-	-	-	-	-	-	19,494,010
Supplies: intensive list	2021 In-person, air	39,213	39,213	-	93,908,607	882,760		
	2021 In-person, car	1,250,220	1,250,220	42,881,325	-	32,426,310		

Supplemental Table 3: Model inputs and assumptions of clinic energy consumption and surface area; Base case and min/max scenario modeling

**Measured Data related to Energy Consumption of in-Person Systems**	**Electricity (kWh/sf/min)**	**Natural Gas (MJ/sf/min)**	**Chilled water (kWh/sf/min)**	**Steam (MJ/sf/min)**	**Room size (sf), assumed**
Base: (Campus Average)	5.47652E-05	0.000103312	7.76368E-06	0.000117187	100
Maximum	6.88927E-05	0.000574177	1.24724E-05	0.000214128	225
Minimum	2.04401E-05	0	0	0	64

Supplemental Table 4: Per-patient Greenhouse Gas emissions at SHC in 2021 by Department. SMP = Stanford Medical Partners, GI = Gastrointestinal

**Department**	kgCO2e/ patient (in person only)	kgCO2e/ patient (virtual only)	GHG reductions
Acute Care Surgery	23.19	0.0233	99.90%
Cancer	25.80	0.0344	99.87%
Cardiovascular	29.99	0.0513	99.83%
Dermatology	16.92	0.0226	99.87%
GI/GI Surgery	25.37	0.0465	99.82%
Gynecology	19.91	0.0479	99.76%
Medical Specialties	23.39	0.0492	99.79%
Neurosciences	37.72	0.0501	99.87%
Ophthalmology	20.15	0.0252	99.87%

Orthopedics	63.85	0.0258	99.96%
Otolaryngology	25.93	0.0429	99.83%
Pain management	23.83	0.0702	99.71%
Plastic Surgery	24.56	0.0292	99.88%
Primary Care	11.54	0.0423	99.63%
Psychiatry	35.91	0.0751	99.79%
Pulmonary & Critical Care	30.30	0.0478	99.84%
SMP Oncology	9.18	0.0276	99.70%
SMP Primary Care & Pediatrics	7.33	0.0311	99.58%
SMP Specialty Care	8.90	0.0317	99.64%
Urology	18.70	0.0390	99.79%
**TOTAL**	20.04	0.0430	99.79%

Supplemental Table 5: Life Cycle Results for 2021 data (cumulative); WECC= Western Electricity Coordinating Council

**Impact category**	**Unit**	**Phone, one minute, WECC grid**	**Video, one minute, WECC grid**	**In person, Car**	**In person, Air**	**TOTAL (2021 SHC Visits)**
Ozone depletion	kg CFC-11 eq	9.20952E-05	0.001844036	2.849211375	2.602209842	5.453357348
Global warming	kg CO2 eq	1,332.07	27,568.70	14,426,785.18	11,411,217.10	25,866,903
Smog	kg O3 eq	59.16748557	1110.417111	834839.8399	1228089.108	2064098.532
Acidification	kg SO2 eq	4.855870369	88.11502952	51025.64683	50095.49919	101214.1169
Eutrophication	kg N eq	10.85541512	220.3741332	25299.53295	10125.96	35656.72249
Carcinogenics	CTUh	0.000190171	0.003448333	1.843533223	0.14201771	1.989189437
Non carcinogenics	CTUh	0.000898494	0.015376862	3.59232697	1.464095831	5.072698157

Respiratory effects	kg PM2.5 eq	3.341351662	74.15252071	8584.59233	3352.933977	12015.02018
Ecotoxicity	CTUe	86675.62949	1491669.186	470255246	24820556.14	496654147
Fossil fuel depletion	MJ surplus	1333.309503	27900.48216	26547185.19	23353168.31	49929587.29

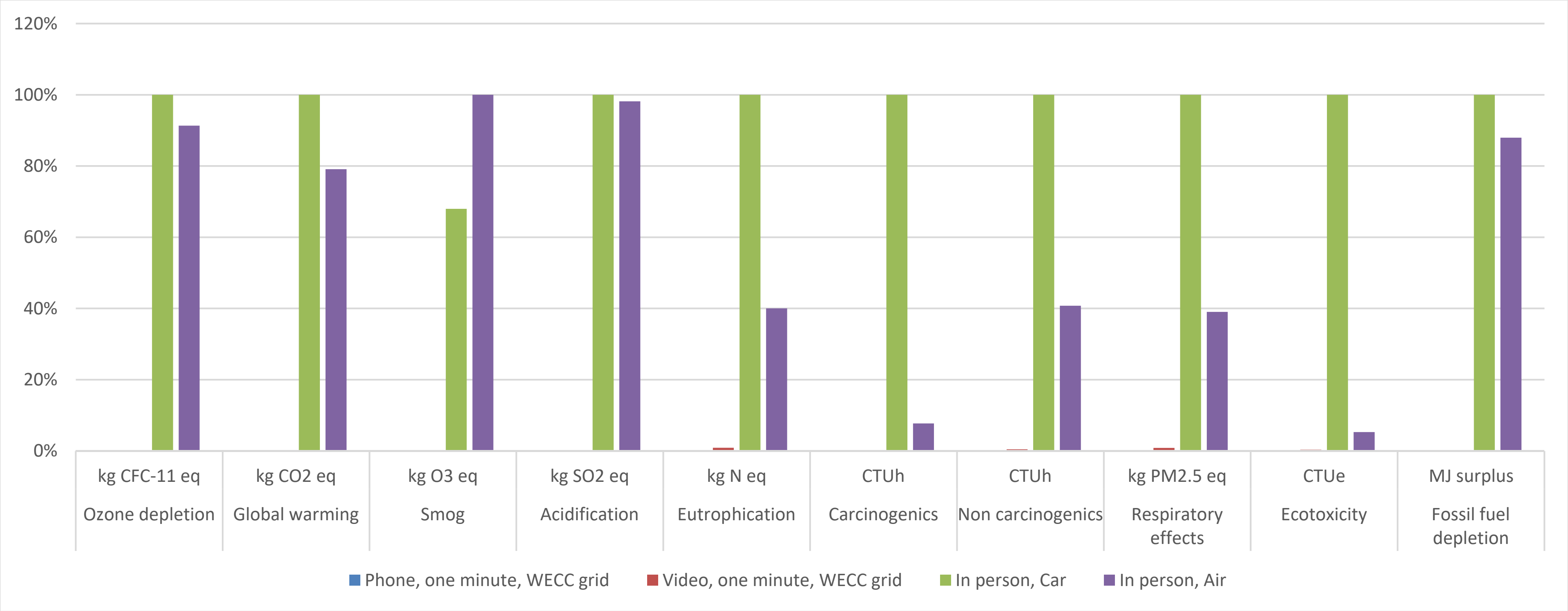

Supplemental Figure 1: Life Cycle Results for Stanford Health Care Clinic Visits; normalized to largest value in each impact category

Supplemental Table 6: Life Cycle Results for 2021 car travel visits (cumulative); WECC= Western Electricity Coordinating Council

Impact category	In-person visit (minimal supplies)	Stanford medical trash (minimal supplies)	Transport, passenger car {RoW}| market for | Cut-off, U	One minute in-person Visit, WECC grid
Ozone depletion	0.0101901	0.000727	2.826965627	0.011328813
Global warming	91,731.47	7,793.13	14,203,972.82	123,287.77
Smog	5298.1457	929.0306	825137.4348	3475.228832
Acidification	472.98291	31.49692	50276.49885	244.6681525
Eutrophication	664.04068	53.41693	23945.3473	636.7280307
Carcinogenics	0.0080544	0.001471	1.825262985	0.008744543
Non carcinogenics	0.1050632	0.009873	3.4486644	0.028725985
Respiratory effects	51.615996	2.710798	8282.304483	247.9610527
Ecotoxicity	1389622.3	790903.1	465241521.7	2833198.922
Fossil fuel depletion	174593.65	6126.335	26172251.35	194213.8522

Supplemental Table 7: Life Cycle Results for 2021 air travel visits (cumulative); WECC= Western Electricity Coordinating Council

Impact category	In-person visit (minimal supplies)	Stanford medical trash (minimal supplies)	Transport, passenger aircraft, short haul {GLO}| market for transport, passenger aircraft, short haul | Cut-off, U	One minute in-person Visit, WECC grid
Ozone depletion	0.00032	2.28E-05	2.601559023	0.000308
Global warming	2,877.15	244.43	11,404,739.19	3,356.33
Smog	166.1757	29.13893	1227799.185	94.60814
Acidification	14.83505	0.987897	50073.0155	6.660741
Eutrophication	20.82756	1.675416	10086.12301	17.33401
Carcinogenics	0.000253	4.61E-05	0.14148088	0.000238
Non carcinogenics	0.003295	0.00031	1.459708835	0.000782
Respiratory effects	1.61893	0.085024	3344.479638	6.750386
Ecotoxicity	43585.34	24806.58	24675034.43	77129.8
Fossil fuel depletion	5476.109	192.1518	23342212.86	5287.195

Supplemental Table 8: Detailed results for GHG emissions in sensitivity analyses (SHC 2021 data); WECC= Western Electricity Coordinating Council

GHG Results, Energy Sources	**baseline (WECC)**	**WECC+solar**	**US avg**	**% difference from baseline**		
In-person visits (car)	14,426,785	14,355,862	14447096.7	0.0%	-0.5%	0.1%
In-person visits (air)	11,411,217	11,409,286	11411770.09	0.0%	0.0%	0.0%
Phone visits	1,332.07	507.63	1,568.18	0.0%	-61.9%	17.7%
Video visits	27,568.70	7,884.88	33,205.86	0.0%	-71.4%	20.4%
In-person TOTAL	25,838,002.38	25,765,148.15	25,858,866.78	0.0%	-0.3%	0.1%
Virtual TOTAL	28,900.77	8,392.52	34,774.04	0.0%	-71.0%	20.3%

"Savings" of virtual system over in-person (if virtual was done in person)	16,926,497	16,900,783.50	16,933,860.99	0.0%	-0.2%	0.0%
If in-person was all cars	45,539,469			76.2%		
**GHG Results, Energy Intensity (HVAC system)**	**Min energy intensity**	**Baseline**	**Max energy intensity**	**% difference from baseline**		
In-person visits (car)	14,320,619	14,426,785	14,793,903	-0.7%	0.0%	2.5%
In-person visits (air)	11,408,327	11,411,217	11,421,211	0.0%	0.0%	0.1%
Phone visits	1,332.07	1,332	1,332.07	0.0%	0.0%	0.0%
Video visits	27,568.70	27,569	27,568.70	0.0%	0.0%	0.0%
In-person TOTAL	25,728,945.90	25,838,002.38	26,215,114.50	-0.4%	0.0%	1.5%
Virtual TOTAL	28,900.77	28,900.77	28,900.77	0.0%	0.0%	0.0%
"Savings" of virtual system over in-person (if virtual was done in person)	16,857,307	16,926,497	17,165,753	-0.4%	0.0%	1.4%
**GHG Results, Intensive Supply List**	**Baseline**	**Max supplies**	**% difference from baseline**			
In-person visits (car)	14,355,862	14,618,550	0.0%	1.8%		
In-person visits (air)	11,411,217	11,417,148	0.0%	0.1%		
Phone visits	1,332	1,332.07	0.0%	0.0%		
Video visits	27,569	27,568.70	0.0%	0.0%		
In-person TOTAL	25,767,078.94	26,035,697.61	0.0%	1.0%	268,618.66	
Virtual TOTAL	28,901	28,900.77	0.0%	0.0%		

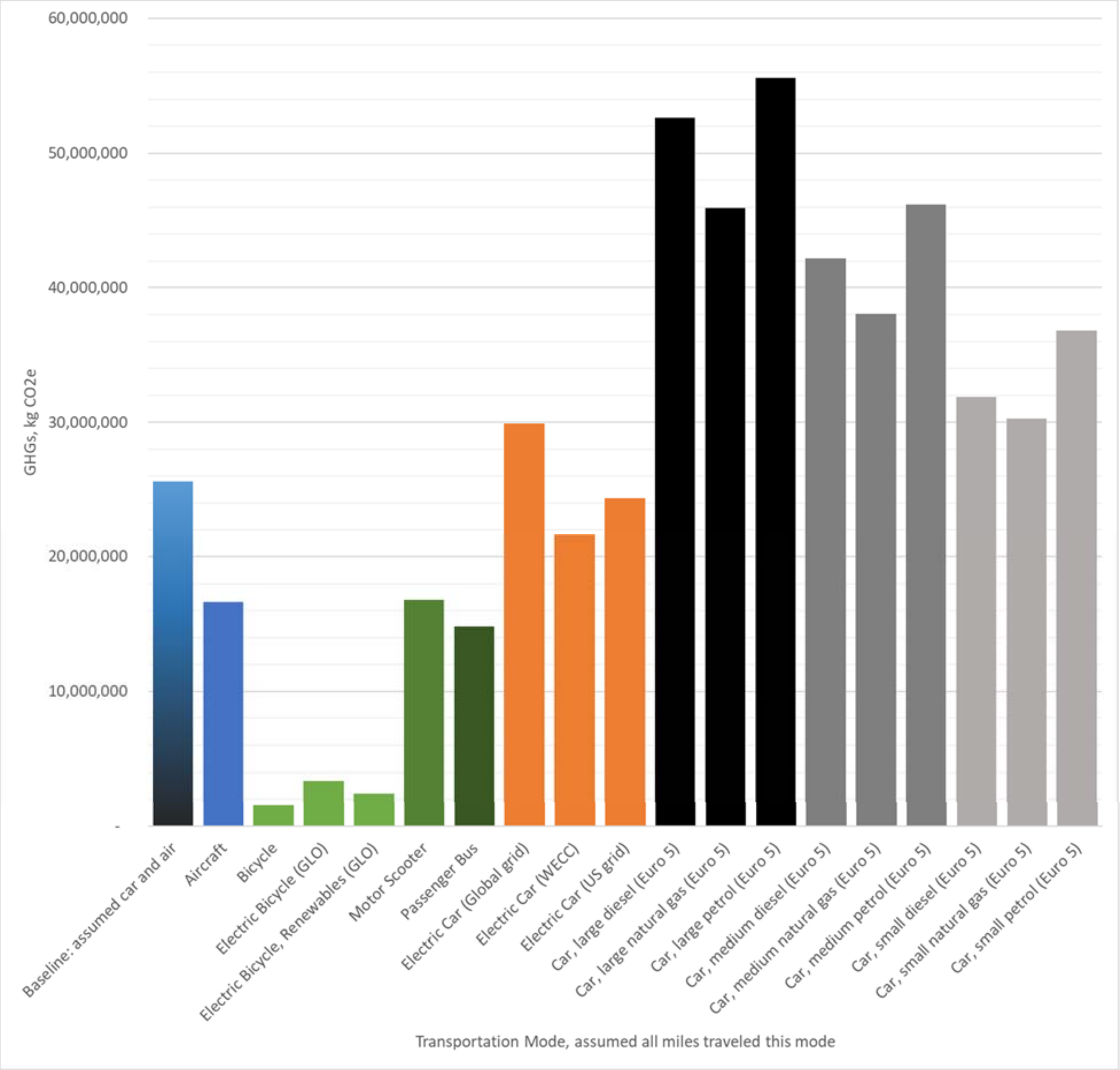

Figure 2: Variation in estimated emissions from patient travel modes, showing only transportation emissions for in-person visits in 2021. GLO=global energy mis, WECC= Western Electricity Coordinating Council or California's grid mix, US avg= United States average grid mix, Euro 5=passenger vehicles conforming to European emissions standards from 2008 (the most recent passenger vehicles available in the Ecoinvent database)

List of Ecoinvent v3.8 unit processes used for Transportation Mode sensitivity analyses:

We assumed one person traveling per visit.

- 1 personkm Transport, passenger, bicycle {GLO}| market for | Cut-off, U (of project Ecoinvent 3 - allocation, cut-off by classification - unit)
- 1 personkm Transport, passenger, electric bicycle {GLO}| market for | Cut-off, U (of project Ecoinvent 3 - allocation, cut-off by classification - unit)
- 1 personkm Transport, passenger, electric bicycle, electricity from renewable energy products {GLO}| market for transport, passenger, electric bicycle, electricity from renewable energy products | Cut-off, U (of project Ecoinvent 3 - allocation, cut-off by classification - unit)
- 1 personkm Transport, passenger, motor scooter {GLO}| market for | Cut-off, U (of project Ecoinvent 3 - allocation, cut-off by classification - unit)
- 1 personkm Transport, regular bus {GLO}| market for | Cut-off, U (of project Ecoinvent 3 - allocation, cut-off by classification - unit)
- 1 km Transport, passenger car, electric {GLO}| market for | Cut-off, U (of project Ecoinvent 3 - allocation, cut-off by classification - unit)
- 1 km Transport, passenger car, large size, diesel, EURO 5 {GLO}| market for | Cut-off, U (of project Ecoinvent 3 - allocation, cut-off by classification - unit)
- 1 km Transport, passenger car, large size, natural gas, EURO 5 {GLO}| market for | Cut-off, U (of project Ecoinvent 3 - allocation, cut-off by classification - unit)
- 1 km Transport, passenger car, large size, petrol, EURO 5 {GLO}| market for | Cut-off, U (of project Ecoinvent 3 - allocation, cut-off by classification - unit)
- 1 km Transport, passenger car, medium size, diesel, EURO 5 {GLO}| market for | Cut-off, U (of project Ecoinvent 3 - allocation, cut-off by classification - unit)
- 1 km Transport, passenger car, medium size, natural gas, EURO 5 {GLO}| market for | Cut-off, U (of project Ecoinvent 3 - allocation, cut-off by classification - unit)
- 1 km Transport, passenger car, medium size, petrol, EURO 5 {GLO}| market for | Cut-off, U (of project Ecoinvent 3 - allocation, cut-off by classification - unit)
- 1 km Transport, passenger car, small size, diesel, EURO 5 {GLO}| market for | Cut-off, U (of project Ecoinvent 3 - allocation, cut-off by classification - unit)
- 1 km Transport, passenger car, small size, natural gas, EURO 5 {GLO}| market for | Cut-off, U (of project Ecoinvent 3 - allocation, cut-off by classification - unit)
- 1 km Transport, passenger car, small size, petrol, EURO 5 {GLO}| market for | Cut-off, U (of project Ecoinvent 3 - allocation, cut-off by classification - unit)
- 1 km Transport, passenger car, electric {WECC}| market for | Cut-off, U (of project Stanford Virtual Visits)
- 1 km Transport, passenger car, electric {US}| market for | Cut-off, U (of project Stanford Virtual Visits)
